# Supplementary material for: The genome sequence of a hoverfly , Neoascia tenur (Harris, 1780) (Diptera: Syrphidae)
Source: Wellcome Open Res. 2026 Jun 11;11:349. [Version 1] doi: 10.12688/wellcomeopenres.26866.1 (PMC13316065; doi:10.12688/wellcomeopenres.26866.1)
Supplement: Peer review history

# Reviewer report: Reviewer response for version 1

**DOI:** 10.21956/wellcomeopenres.29585.r158792

**Published:** 2026-07-09

**Authors:** Xin Liu

**Affiliations:** 1 Beijing Genomics Institute, Beijing, Beijing, China

This is a typical Data Note article. We appreciate the continued contribution of the Darwin Tree of Life (DToL) initiative to genome data generation and research. Liam et al. report the sequencing, genome assembly, and basic data interpretation of the hoverfly species *Neoascia tenur*. The methodology follows the standard DToL pipeline, with sample collection, data generation, assembly, and evaluation all consistent with previous Data Notes. The approach is overall reliable, and the assembly quality is excellent. The data produced here will be valuable for downstream comparative genomics and other studies.

 I have the following suggestions to help improve the clarity and rigor of the manuscript:

**1. The sex and origin of the Hi-C sequencing sample**

 Although the methods are clearly described, the sex of the Hi-C sample is not explicitly stated. Is it assumed to be the same as the HiFi data, i.e., derived from a female individual? If it is indeed female, the statement that sex chromosomes were "not identified" seems unnecessary.

 a. Given that the authors have raised this point, I suggest providing photographs of both samples (at minimum, the idNeoTenu1 specimen) with clear annotations on the key morphological features used for sex and species identification, particularly since barcode-based species identification was already employed.

 b. Regarding the claims about sex chromosomes and the fourth chromosome being a dot chromosome, experimental validation (e.g., karyotyping) or citation of relevant literature would strengthen the argument. This is especially important because the Hi-C and HiFi samples are not identical, and individual variation may exist.

 c. To assess inter-individual differences, I suggest generating low-coverage WGS data from the Hi-C sample and performing k-mer analysis or alignment against the HiFi assembly to evaluate the degree of divergence between the two individuals.

**2. Discrepancy between assembled genome size and k-mer-based estimates**

 While deviations between k-mer-based estimates and final assembly sizes are common, the predicted heterozygosity of 3.57%—combined with the notable size discrepancy—warrants closer attention to potential assembly issues caused by high heterozygosity. The final assembly is 491 Mb with less than 3% of sequences unanchored to chromosomes. Given the genome size discrepancy and high heterozygosity mentioned above, I recommend that the authors validate the assembly by examining read alignment depth distributions to identify problematic regions. Although MerquryFK was used for k-mer-based assessment, the current results do not readily allow evaluation of whether the k-mer depth distribution matches expectations, nor do they permit region-specific inspection of k-mer depth. I suggest supplementing the analysis with HiFi read alignments or additional short-read alignments to verify whether high heterozygosity has introduced redundant sequences or misassemblies.

Are sufficient details of methods and materials provided to allow replication by others?

Yes

Is the rationale for creating the dataset(s) clearly described?

Yes

Are the datasets clearly presented in a useable and accessible format?

Yes

Are the protocols appropriate and is the work technically sound?

Yes

Reviewer Expertise:

genomics

I confirm that I have read this submission and believe that I have an appropriate level of expertise to confirm that it is of an acceptable scientific standard, however I have significant reservations, as outlined above.

# Reviewer report: Reviewer response for version 1

**DOI:** 10.21956/wellcomeopenres.29585.r158800

**Published:** 2026-06-29

**Authors:** Jia chang

**Affiliations:** 1 The University of Melbourne, Melbourne, Victoria, Australia

This article presents a high-quality chromosome-level genome assembly of *Neoascia tenur* generated as part of the Darwin Tree of Life Project. The assembly was produced using PacBio HiFi sequencing combined with Hi-C scaffolding and achieves excellent assembly statistics. The sequencing strategy, assembly workflow, and public data deposition are consistent with current standards for reference genome production.

 The sequencing strategy, assembly pipeline, scaffolding, and quality assessment are technically sound and follow current best practices. However, the contamination screening results are insufficiently reported, and additional clarification regarding ASCC outputs would improve confidence in the final assembly.

 Comments:

 1. Contamination screening should be described in greater detail. Contamination screening is a critical but challenging step in reference genome assembly. The manuscript states that the assembly was decontaminated using the Assembly Screen for Cobionts and Contaminants (ASCC) pipeline, but no quantitative results are presented regarding the contamination screening process.

 2. The authors should briefly state whether a chromosome number of four is expected for *Neoascia tenur *or related *Syrphidae*, and provide appropriate references if such information is available. This would place the chromosome-level assembly into a clearer biological context.

 3. The manuscript states that chromosome 4 is interpreted as a dot chromosome. It would be helpful to provide a brief citation or comparative evidence supporting this assignment within *Syrphidae *or *Diptera*.

Are sufficient details of methods and materials provided to allow replication by others?

Yes

Is the rationale for creating the dataset(s) clearly described?

Yes

Are the datasets clearly presented in a useable and accessible format?

Yes

Are the protocols appropriate and is the work technically sound?

Partly

Reviewer Expertise:

genomics; population genetics; endosymbionts

I confirm that I have read this submission and believe that I have an appropriate level of expertise to confirm that it is of an acceptable scientific standard.

# Reviewer report: Reviewer response for version 1

**DOI:** 10.21956/wellcomeopenres.29585.r158795

**Published:** 2026-06-26

**Authors:** Matsapume Detcharoen

**Affiliations:** 1 Prince of Songkla University, Hat Yai, Thailand

This manuscript reports a chromosome-level genome assembly for Neoascia tenur plus a 16.31 kb mitochondrial genome.

 A major strength is the high technical quality and transparency of the genome assembly, supported by PacBio HiFi and Hi-C data, manual curation, strong scaffold contiguity, high BUSCO completeness, high consensus quality, making the resource reproducible and useful for comparative genomics of hoverflies and related Diptera.

Are sufficient details of methods and materials provided to allow replication by others?

Yes

Is the rationale for creating the dataset(s) clearly described?

Yes

Are the datasets clearly presented in a useable and accessible format?

Yes

Are the protocols appropriate and is the work technically sound?

Yes

Reviewer Expertise:

Molecular ecology

I confirm that I have read this submission and believe that I have an appropriate level of expertise to confirm that it is of an acceptable scientific standard.
